# Sexually and non‐sexually transmitted infections and the risk of prostate cancer: Results from the EPICAP study

**DOI:** 10.1002/cam4.6841

**Published:** 2024-01-04

**Authors:** Melissa Sawaya, Emilie Cordina‐Duverger, Pierre‐Jean Lamy, Xavier Rébillard, Brigitte Trétarre, Florence Menegaux

**Affiliations:** ^1^ Université Paris‐Saclay, UVSQ, Inserm, Gustave Roussy, CESP Villejuif France; ^2^ Service de recherche clinique, Clinique Beau Soleil Montpellier France; ^3^ Service Urologie, Clinique Beau Soleil Montpellier France; ^4^ Registre des Tumeurs de l'Hérault, EA 2415, ICM Montpellier France; ^5^ Center for Epidemiology and Research in Population Health (CERPOP) Toulouse France

**Keywords:** cancer prevention, cancer risk factors, epidemiology, prostate cancer

## Abstract

**Introduction:**

Prostate cancer (PCa) is by far the most common type of cancer among men in western countries. However, relatively little is known about its etiology despite the high morbidity and mortality. It has been suggested that chronic inflammation may be involved in prostate carcinogenesis. We investigated the role of sexually and non‐sexually transmitted infections in prostate cancer risk with a specific interest in the aggressive types.

**Methods:**

We used data from epidemiological study of prostate cancer (EPICAP), a population‐based case–control study. A total of 819 incident cases and 879 controls were interviewed face‐to‐face using a standardized questionnaire gathering information on known or suspected risk factors of prostate cancer and personal history of specific sexually and non‐sexually transmitted infections: gonorrhea, syphilis, trichomonas, herpes, mononucleosis, Epstein–Barr virus, varicella‐zoster, and dengue. Odds ratios (OR) and their 95% confidence interval were estimated using multivariate unconditional logistic regression.

**Results:**

There was no significant association between gonorrhea (OR: 0.90, 95% CI: 0.61–1.33), trichomonas (OR: 0.74, 95% CI: 0.27–2.07), genital herpes (OR: 0.69, 95% CI: 0.38–1.27), and the risk of prostate cancer. No association emerged for overall sexually transmitted bacterial and viral infections (OR 1.05, 95% CI: 0.86–1.29) and overall non‐sexually transmitted viral infections (OR 1.11, 95% CI: 0.90–1.35) and the risk of prostate cancer.

**Conclusion:**

Our results showed that sexually or non‐sexually transmitted infections, either bacterial or viral, were not associated to prostate cancer. Therefore, further investigation is needed to help advance our understanding of the role of chronic inflammation in the etiology of prostate cancer, with a particular focus on its most aggressive types.

## 1 INTRODUCTION

Prostate cancer (PCa) is by far the most common type of cancer among men in western countries, with an estimate of 1,414,295 new cases and roughly 375,000 deaths worldwide in 2020.^1^ More than 60,000 new cases in France were diagnosed, with approximately 9000 deaths in 2020, making it the second leading cause of cancer‐related mortality.^2^, ^3^

Relatively little is known about the etiology of prostate cancer despite the high morbidity and mortality rates, where only advanced age, ethnicity, and family history of prostate cancer are well‐established non‐modifiable risk factors.^4^ However, according to studies done on migrants, Asian men living in the United States had a much higher incidence rate of prostate cancer compared to those living in their native countries, suggesting the role of lifestyle and environmental factors.^5^

Chronic inflammation has been associated with a wide range of malignancies, such as liver, kidney, and lung, via a specific infectious or environmental agent.^6^, ^7^ It has been suggested through several epidemiological studies that inflammation of the prostate may also be involved in the development of prostate carcinogenesis.^8^, ^9^ In fact, inflammatory infiltrates located near areas of proliferative inflammatory atrophy (PAI) and prostatic intraepithelial neoplasia (PIN), considered precancerous prostate lesions, strengthen the hypothesis of a possible link between chronic inflammation and prostate cancer.^9^, ^10^

While the cause behind prostatic inflammation remains unclear, several inflammation‐related factors might be potential agents contributing to the onset of prostate cancer.^9^ Notably, infectious agents, including bacteria and viruses, have been observed to infect the prostate and trigger an inflammatory response and therefore are potential contributors to prostate carcinogenesis.^9^ While infectious agents can be classified into non‐sexually and sexually transmitted infections (STIs), it is worth noting that the latter has received more extensive attention over the years compared to the former. For instance, the role of Neisseria gonorrhea, Chlamydia trachomatis, Trichomonas vaginalis, syphilis, herpes simplex virus (HSV), herpes papillomavirus (HPV) 16 and 18, cytomegalovirus (CMV), and Epstein–Barr virus (EBV) in the occurrence of prostate cancer has been studied, yet the findings were inconsistent.^11^ Case–control studies^12^, ^13^, ^14^, ^15^ and prospective cohort studies^16^, ^17^, ^18^, ^19^ mainly focused on gonorrhea, syphilis, genital herpes and did not find any association with a history of gonorrhea,^12^, ^13^, ^14^, ^18^ syphilis,^12^, ^13^, ^14^, ^15^ trichomonas,^14^ or genital herpes,^12^, ^14^, ^18^, ^20^ and prostate cancer. Similarly, investigations into blood‐borne viral infections such as hepatitis B and C known for their liver‐related impact, yielded inconsistent results.^21^, ^22^ Given the often asymptomatic nature of STIs, additional resources have been established, like blood collection and serological detection, allowing the prospective examination of STIs and their associated risk with prostate cancer.^23^ The role of HSV‐1 and HSV‐2 viruses,^24^, ^25^ CMV,^25^, ^26^ Trichomonas vaginalis,^27^, ^28^, ^29^ and EBV^30^ were studied through serological essays with varying results.

Among non‐sexually transmitted infections, significant attention has been directed toward genitourinary infections, particularly prostatitis^31^ and various other genitourinary infections, such as urethritis, orchitis, epididymitis, and pyelonephritis.^32^, ^33^ Herpes zoster, on the other hand, often known as shingles, is caused by the reactivation of the varicella‐zoster virus (VZV) previously latent after primary infection with VZV has been suggested to directly affect the development of cancer, including prostate cancer.^34^ However, the existing body of research on this topic is limited and inconclusive.^35^, ^36^ Importantly, and beyond varicella‐zoster, it is noteworthy to mention that non‐sexually transmitted infections, such as polio, tuberculosis, and dengue, have yet to be explored in relation to prostate cancer.

To date, very few studies have considered the aggressiveness of cancer while studying chronic inflammation and/or infection.^14^, ^18^

In this context, the main objective of this study was to examine the association of sexually and non‐sexually transmitted infections, whether bacterial or viral, in the occurrence of prostate cancer, with a particular interest on the aggressive types using data from the EPICAP study.

## 2 METHODS

### 2.1 Study population

EPICAP (Epidemiological study of prostate cancer) is a population‐based case–control study conducted in the department of Hérault located in the south of France between 2012 and 2014 to investigate the role of environmental and genetic factors in the occurrence of prostate cancer. Details of the EPICAP objectives and study design have been previously described.^37^ Eligible cases were men newly diagnosed with prostate cancer between 2012 and 2013 who were under the age of 75 and living in the department of Hérault at the time of diagnosis. All cases included in the study were histologically confirmed. The identification of cases was done by clinical research nurses, specifically trained for the study, by active search in all public and private cancer care centers of the department. Controls were randomly selected from the general population in the department of Hérault that were not diagnosed with prostate cancer and living in the same department at the time of inclusion. To achieve frequency‐matching, quotas by age were established as a preliminary to yield a control group similar to the case group in terms of age (5‐year age group). Quotas on socioeconomic status were also set a priori to yield a control group comparable to the general male population of the same age in the department of Hérault.

In total, EPICAP had 1098 prostate cancer cases, and 1109 population‐based controls that were eligible, of which 819 cases and 879 controls were included, with a participation rate of 75% and 79%, respectively.

### 2.2 Data collection

Cases and controls were interviewed face‐to‐face by a well‐trained clinical research nurse, using a standardized computer‐assisted questionnaire (CAPI). Information on sociodemographic and lifestyle characteristics (Tobacco, alcohol consumption, and physical activity), occupational and residential history, personal medical history and drug use, family history of prostate cancer, and anthropomorphic measurements (weight, height, abdominal, and hip perimeter) were collected.

For tobacco consumption, men were asked during the interview whether they are smokers, non‐smokers, or former smokers. Therefore, it was classified into three categories: former, current, and non‐smoker.

For alcohol consumption, men were asked during the interview whether they drink or not. Therefore, it was classified into two categories: drinkers and non‐drinkers.

For physical activity it was assessed using Metabolic Equivalent Task (MET‐hour/week/year) for each activity practiced for a minimum of 1 h per week over a year. The variable was then later categorized into quartiles based on calculations from the control population.

Important health metrics, including abdominal circumference and body mass index (BMI) were measured by a clinical research nurse.

For non‐steroidal anti‐inflammatory drugs it was categorized into users and non‐users.

Self‐reporting of a personal history of sexually or non‐sexually transmitted infections, whether bacterial (gonorrhea, syphilis, trichomonas, tuberculosis) or viral (herpes, Epstein–Barr, zoster herpes, dengue, polio, viral hepatitis), and their conditions such as urethritis and mononucleosis were collected. In addition, the number of infections for each type during their lifetime and age/year at the time of infection was also collected.

Clinical medical information for each case, such as Gleason scores and prostate specific antigen (PSA), were extracted from the patient's medical records at the time of diagnosis and validated by the Hérault cancer registry.

### 2.3 Statistical analysis

All analyses were performed using the statistical analysis software SAS (9.4 version).

Personal history of each sexually or non‐sexually transmitted bacterial or viral infection and their conditions (gonorrhea, syphilis, trichomonas, urethritis, tuberculosis, zoster herpes, mononucleosis, Epstein–Barr, zona, polio, dengue, herpes genital and non‐genital, mononucleosis, viral hepatitis) were defined separately by ever having an infection before the reference date (date of diagnosis for the cases and date of interview for the controls).

Similar types of infections were combined in different groups to have an overall view of the association between all types of infections and prostate cancer.

*Sexually Transmitted Bacterial Infections* were defined by having at least one type of bacterial infection (gonorrhea, syphilis, trichomonas) before the reference date.

*Viral infections* were defined by having at least one type of sexually or non‐sexually transmitted viral infection or their condition (zoster herpes, polio, dengue, herpes genital and non‐genital, Epstein–Barr, viral hepatitis, mononucleosis) before the reference date.

*Sexually transmitted viral infections* were defined by having at least one type of sexually transmitted viral infection or their condition (mononucleosis, herpes, Epstein–Barr) before the reference date.

*Sexually transmitted bacterial & viral infections* were defined by having one type of sexually transmitted infection or their conditions before the reference date (gonorrhea, syphilis, trichomonas, urethritis, mononucleosis, Epstein–Barr, herpes genital and non‐genital, and mononucleosis) or having two or more types of infections before the reference date.

*Overall infections* were defined by having one type of infection or their conditions (bacterial or viral) or having two or more types of infections before the reference date.

Regarding the reference group, each was defined as having no personal history of the infection or conditions concerned in each group except for all infections, where the reference group is defined as never having any type of infection or condition in their lifetime.

Odds ratios (OR) and their 95% confidence intervals (95% CI) were computed using unconditional logistic regression to investigate the association between sexually and non‐sexually transmitted infections and the risk of prostate cancer.

The analysis was systematically adjusted to the non‐modifiable risk factors: age, ethnicity (Caucasians, others), and family history of prostate cancer in first‐degree relatives as well as potential confounding factors such as education, physical activity, non‐steroidal anti‐inflammatory drugs (NSAIDs), and waist circumference (WC).

Logistic regression models were performed to assess the relationship based on the aggressiveness of prostate cancer (PCa) using the Gleason score at the time of diagnosis. For non‐aggressive or low‐grade cancer, the Gleason score was ≤7 [including 3 + 4], which is considered a low or intermediate score; for aggressive or high‐grade cancer, the Gleason score was ≥7 [including 4 + 3], which is regarded as a high score.

## 3 RESULTS

The characteristics of prostate cancer cases and controls are presented in Table 1. Among cases, 183 (22.7%) were classified as aggressive or high‐grade cancer. The study population was predominantly of Caucasian origin, with 97.1% among cases and 97.7% among controls. Age in a five‐year group (*p* = 0.144), education level (*p* = 0.609), lifestyle factors such as smoking (*p* = 0.288), alcohol consumption (*p* = 0.591), physical activity (*p* = 0.11), and anthropomorphic measurements such as Body Mass Index (*p* = 0.534) and waist circumference (*p* = 0.086) were similarly distributed between cases and controls. Non‐steroidal anti‐inflammatory drugs were more frequent among controls than in cases (*p* = 0.043). As expected, the family history of prostate cancer in first‐degree relatives was more frequent in cases (22%) than in controls (9%) (*p* < 0.0001).

**TABLE 1:** Study population characteristics of epidemiological study of prostate cancer.

	Cases *n* = 819 (%)	Controls *n* = 879 (%)	*p*‐Value
Gleason score			
Low‐grade cancer ≤7 (3 + 4)	623 (77.3)	–	
High‐grade cancer ≥7 (4 + 3)	183 (22.7)	–	
Age (years)			
<55	48 (5.9)	59 (6.7)	0.144
[55–60]	99 (12.1)	99 (11.2)	
[60–65]	217 (26.5)	201 (22.9)	
[65–70]	274 (33.5)	285 (32.4)	
≥70	181 (22.1)	235 (26.7)	
Ethnic origin			
Caucasian	795 (97.1)	859 (97.7)	0.396
Other	24 (2.9)	20 (2.3)	
Family history of prostate cancer			
No	633 (77.8)	799 (91.2)	<0.001
Yes	181 (22.2)	77 (8.8)	
Education			
Primary	179 (21.9)	193 (22.0)	0.609
Secondary	380 (46.4)	425 (48.4)	
University	260 (31.8)	260 (29.6)	
Body mass index (kg/m^2^)			
<25 – normal	231 (28.5)	248 (29.1)	0.534
[25–30] – overweight	399 (49.1)	397 (46.6)	
≥30 – obese	182 (22.4)	207 (24.3)	
Waist circumference (cm)			
≤94	209 (25.9)	254 (29.6)	0.086
>94	599 (74.1)	603 (70.4)	
Smoking status			
Never	240 (29.3)	246 (28)	0.288
Former	455 (55.6)	476 (54.2)	
Current	123 (15.0)	157 (17.9)	
Alcohol consumption			
No	72 (8.8)	84 (9.6)	0.591
Yes	746 (91.2)	795 (90.4)	
Physical activity (MET^a^‐h/wk/yr)			
No^b^	177 (20.1)	191 (23.3)	0.11
<6.25	174 (19.8)	151 (18.4)	
6.25–13.0	174 (19.8)	138 (16.8)	
13.0–24.15	175 (19.9)	147 (17.9)	
≥24.15	174 (19.8)	187 (22.8)	
Non‐steroidal anti‐inflammatory drugs			
No	596 (73.0)	593 (68.6)	0.043
Yes	220 (27.0)	272 (31.5)	

^a^ MET: metabolic equivalent task.

^b^ No: Less than 1 h/week during at least 1 year.

Associations between sexually transmitted bacterial infections (gonorrhea, trichomonas, and syphilis) and prostate cancer risk in all its grades are shown in Table 2. Having a history of gonorrhea (OR: 0.90, CI: 0.61–1.33), trichomonas (OR: 0.74, CI: 0.27–2.07), or syphilis (OR: 0.39, CI: 0.11–1.46) was not associated with the risk of prostate cancer, either for low‐grade or high‐grade cancer in all three bacterial infections. Neither bacterial infections (OR: 0.85 CI: 0.59–1.22) nor their conditions (OR: 0.87 CI: 0.62–1.21) were associated with the risk of prostate cancer in all its grades.

**TABLE 2:** Associations of bacterial infections and prostate cancer risk.

	Controls	Cases					
	*n* = 586 (%)	All		Low‐grade cancer^b^		High‐grade cancer^c^	
		*n* = 591 (%)	OR^a^ (95% CI)	*n* = 623 (%)	OR^a^ (95% CI)	*n* = 183 (%)	OR^a^ (95% CI)
Sexually transmitted bacterial infections^d^							
No	792 (90.9)	750 (92.4)	1.00 Reference	568 (92.1)	1.00 Reference	170 (93.4)	1.00 Reference
Yes	79 (9.1)	62 (7.6)	0.85 (0.59–1.22)	49 (7.9)	0.85 (0.57–1.25)	12 (6.6)	0.83 (0.44–1.58)
Gonorrhea							
No	804 (92.3)	759 (93.2)	1.00 *Reference*	574 (92.7)	1.00 *Reference*	173 (95.1)	1.00 *Reference*
Yes	67 (7.7)	55 (6.8)	0.90 (0.61–1.33)	45 (7.3)	0.93 (0.62–1.41)	9 (4.9)	0.73 (0.35–1.52)
Trichomonas							
No	854 (98.8)	803 (99.1)	1.00 *Reference*	613 (99.5)	1.00 *Reference*	177 (97.8)	1.00 *Reference*
Yes	10 (1.2)	7 (0.9)	0.74 (0.27–2.07)	3 (0.5)	0.31 (0.07–1.53)	4 (2.2)	2.53 (0.76–8.39)
Syphilis							
No	867 (99.0)	816 (99.6)	1.00 *Reference*	621 (99.7)	1.00 *Reference*	182 (99.5)	1.00 *Reference*
Yes	9 (1.0)	3 (0.4)	0.39 (0.11–1.46)	2 (0.3)	0.37 (0.08–1.73)	1 (0.5)	0.51 (0.06–4.15)
Urethritis							
No	836 (97.0)	786 (96.8)	1.00 *Reference*	601 (96.9)	1.00 *Reference*	173 (96.7)	1.00 *Reference*
Yes	26 (3.0)	26 (3.2)	1.04 (0.58–1.88)	19 (3.1)	0.88 (0.46–1.69)	6 (3.4)	1.25 (0.49–3.19)
Tuberculosis							
No	835 (95.2)	774 (94.6)	1.00 *Reference*	589 (94.5)	1.00 *Reference*	172 (94.5)	1.00 *Reference*
Yes	42 (4.8)	44 (5.4)	1.11 (0.70–1.74)	34 (5.5)	1.14 (0.70–1.86)	10 (5.5)	1.07 (0.51–2.23)

^a^ ORs adjusted for age, family history of prostate cancer, ethnicity, physical activity, NSAIDs, waist circumference, education.

^b^ Gleason ≤7 (3 + 4).

^c^ Gleason ≥7 (4 + 3).

^d^ Bacterial Infections: trichomonas, syphilis, gonorrhea.

Associations between sexually (genital herpes, mononucleosis, Epstein–Barr) and non‐sexually transmitted viral infections (zoster herpes, dengue, herpes labialis, polio, viral hepatitis) and prostate cancer risk in all its grades are shown in Table 3. We did not find any association between having a history of zoster herpes (OR:1.04 CI: 0.77–1.41), dengue (OR:0.92 CI: 0.42–2.03), herpes labialis (OR: 1.21 CI 0.97–1.50), genital herpes (OR: 0.69 CI: 0.38–1.27) Epstein–Barr (OR: 0.92 CI: 0.46–1.86), or viral hepatitis (OR:1.18 CI:0.85–1.65), and the risk of prostate cancer. Similarly, the association did not change as well among low‐grade and high‐grade cancer in all of the viral infections. The overall viral infections group was also not associated to PCa, whether for all viruses (OR: 1.11 CI: 0.90–1.35) or only for sexually transmitted viral infections (OR: 1.12 CI: 0.91–1.38), even for low‐grade and high‐grade PCa. Finally, the association between overall bacterial and viral infections combined and prostate cancer is shown in Table 4. For sexually transmitted bacterial and viral infections combined, there was no association in all prostate cancer cases (OR: 1.05 CI: 0.86–1.29). In regard to the overall infections having one infection, whether bacterial or viral, in their lifetime yielded an OR of 1.07 (CI: 0.86–1.34), and it increased to OR: 1.17 (CI: 0.88–1.56) when having more than one type of infection, though it was not significantly associated with prostate cancer even for low‐grade and high‐grade cancer.

**TABLE 3:** Associations of viral infections and prostate cancer risk.

	Controls	Cases					
	*n* = 879 (%)	All		Low‐grade cancer^b^		High‐grade cancer^c^	
		*n* = 819 (%)	OR^a^ (95% CI)	*n* = 623 (%)	OR^a^ (95% CI)	*n* = 183 (%)	OR^a^ (95% CI)
Viral infections^d^							
No	443 (52.7)	408 (50.9)	1.00 Reference	305 (50.1)	1.00 Reference	95 (53.1)	1.00 Reference
Yes	398 (47.3)	393 (49.1)	1.11 (0.90–1.35)	304 (49.9)	1.16 (0.93–1.44)	84 (46.9)	1.02 (0.73–1.42)
Zoster herpes							
No	761 (87.6)	713 (87.5)	1.00 Reference	542 (87.0)	1.00 Reference	158 (87.6)	1.00 Reference
Yes	108 (12.4)	102 (12.5)	1.04 (0.77–1.41)	80 (13.0)	1.11 (0.80–1.53)	22 (12.2)	0.96 (0.58–1.58)
Polio							
No	872 (99.5)	816 (99.8)	1.00 *Reference*	622 (99.8)	1.00 *Reference*	181 (99.5)	1.00 *Reference*
Yes	4 (0.5)	2 (0.2)	1.46 (0.20–10.58)	1 (0.2)	1.18 (0.11–13.21)	1 (0.6)	2.51 (0.21–30.44)
Dengue							
No	858 (98.3)	803 (98.4)	1.00 Reference	610 (98.2)	1.00 Reference	181 (98.9)	1.00 Reference
Yes	15 (1.7)	13 (1.6)	0.92 (0.42–2.03)	11 (1.8)	1.03 (0.45–2.34)	2 (1.1)	0.62 (0.13–2.88)
Viral hepatitis							
No	780 (90.7)	725 (89.2)	1.00 Reference	554 (89.6)	1.00 Reference	159 (87.4)	1.00 Reference
Yes	80 (9.3)	88 (10.8)	1.18 (0.85–1.65)	64 (10.4)	1.13 (0.7–1.62)	23 (12.6)	1.56 (0.93–2.62)
Sexually transmitted viral infections^e^							
No	575 (66.9)	524 (64.9)	1.00 Reference	392 (63.7)	1.00 Reference	123 (68.3)	1.00 Reference
Yes	284 (33.1)	284 (35.2)	1.12 (0.91–1.38)	223 (36.3)	1.17 (0.93–1.47)	57 (31.7)	0.98 (0.69–1.40)
Genital Herpes							
No	847 (96.8)	793 (97.3)	1.00 Reference	602 (97.0)	1.00 Reference	178 (97.8)	1.00 Reference
Yes	28 (3.2)	22 (2.7)	0.69 (0.38–1.27)	18 (3.0)	0.73 (0.38–1.39)	4 (2.2)	0.62 (0.21–1.83)
Epstein–Barr							
No	849 (98.0)	786 (97.9)	1.00 *Reference*	599 (97.9)	1.00 *Reference*	174 (97.8)	1.00 *Reference*
Yes	17 (2.0)	17 (2.1)	0.92 (0.46–1.86)	13 (2.1)	0.89 (0.42–1.90)	4 (2.3)	1.08 (0.34–3.41)
Herpes labialis							
No	615 (70.9)	555 (68.1)	1.00 Reference	416 (67.2)	1.00 Reference	130 (71.0)	1.00 Reference
Yes	253 (29.1)	260 (31.9)	1.21 (0.97–1.50)	203 (32.8)	1.26 (0.99–1.59)	53 (29.0)	1.05 (0.73–1.52)

^a^ ORs adjusted for age, family history of prostate cancer, ethnicity, physical activity, NSAIDs, waist circumference, education.

^b^ Gleason ≤7 (3 + 4).

^c^ Gleason ≥7 (4 + 3).

^d^ Viral infections: herpes zoster, dengue, Herpes labial & genital, mononucleosis, Epstein–Barr, polio, viral hepatitis.

^e^ Sexually transmitted viral infections: Herpes labial & genital, Epstein–Barr.

**TABLE 4:** Overall association of sexually and non‐sexually transmitted infections and prostate cancer risk.

	Controls	Cases					
	*n* = 879 (%)	All		Low‐grade cancer^b^		High‐grade cancer^c^	
		*n* = 819 (%)	OR^a^ (95% CI)	*n* = 623 (%)	OR^a^ (95% CI)	*n* = 183 (%)	OR^a^ (95% CI)
Sexually transmitted bacterial & viral infections^d^							
No	511 (60.1)	476 (59.3)	1.00 Reference	358 (58.5)	1.00 Reference	111 (62.4)	1.00 Reference
Yes	340 (40.0)	327 (40.7)	1.05 (0.86–1.29)	254 (401.5)	1.06 (0.85–1.32)	67 (37.6)	0.97 (0.69–1.38)
1	287 (33.7)	277 (34.5)	1.05 (0.85–1.30)	216 (35.2)	1.07 (0.85–1.35)	55 (30.9)	0.93 (0.65–1.35)
≥2	53 (6.2)	50 (6.2)	1.04 (0.67–1.59)	38 (6.2)	0.97 (0.61–1.56)	12 (6.7)	1.23 (0.62–2.43)
All infections^e^							
No	383 (45.6)	351 (43.9)	1.00 Reference	264 (43.4)	1.00 Reference	81 (45.5)	1.00 Reference
Yes	457 (54.4)	448 (56.1)	1.10 (0.90–1.35)	344 (56.6)	1.11 (0.89–1.38)	97 (54.5)	1.07 (0.77–1.50)
1	317 (37.7)	305 (38.2)	1.07 (0.86–1.34)	232 (37.7)	1.07 (0.84–1.35)	66 (37.1)	1.03 (0.71–1.49)
≥2	140 (16.7)	143 (17.9)	1.17 (0.88–1.56)	112 (18.4)	1.21 (0.89–1.64)	31 (17.4)	1.18 (0.73–1.89)

^a^ ORs adjusted for age, family history of prostate cancer, ethnicity, physical activity, NSAIDs, waist circumference, education.

^b^ Gleason ≤7 (3 + 4).

^c^ Gleason ≥7 (4 + 3).

^d^ Sexually transmitted bacterial & viral infections: Herpes labial & genital, mononucleosis, Epstein–Barr, trichomonas, syphilis, gonorrhea, urethritis.

^e^ All infections: Herpes labial & genital, mononucleosis, Epstein–Barr, trichomonas, syphilis, gonorrhea, Polio, viral hepatitis, dengue, zona, urethritis, tuberculosis.

## 4 DISCUSSION

Our findings are based on a large, carefully designed population‐based case–control study conducted to address environmental and genetic factors in prostate cancer risk with a specific interest on the aggressive types of prostate cancer.

Our study yielded no evidence of an association between personal history of both sexually and non‐sexually transmitted bacterial and viral infections and the risk of developing prostate cancer. These findings remained consistent even when analyzing the aggressiveness of prostate cancer cases. Therefore, infections may not play a significant role in influencing prostate cancer risk.

Chronic inflammation has been linked to several types of human malignancies. Around 20% of malignancies are caused by chronic infection or chronic inflammatory states. Within the prostate, chronic inflammation may be triggered by infectious agents known as inflammation‐related factors that eventually contribute to the onset of prostate cancer.^8^, ^9^ Data from meta‐analyses^38^, ^39^ are supportive of the role of STIs in the occurrence of prostate cancer. A pooled analysis based on 17 studies were in favor of the role of STI in the occurrence of prostate cancer with an SRR = 1.19 (95% CI: 1.05–1.92).^11^ Similar results were observed in another meta‐analysis yielding an OR = 1.30 (95% CI: 1.30–1.61).^39^ When overall STIs were considered together in studies based on self‐reporting of STIs, two studies^40^, ^41^ found a possible link yielding an OR = 1.7 (95% CI: 1.1–2.5) and OR = 1.68 (95% CI: 1.05–2.7), respectively, and another two^12^, ^18^ found no association yielding an OR = 1.0 (95% CI: 0.7–1.5) and OR = 1.02 (95% CI: 0.91–1.15), respectively, between STIs and prostate cancer which was similar to our results. Our null findings were also consistent with studies that focused on particular types of STIs, such as gonorrhea,^12^, ^16^, ^17^, ^18^ syphilis,^12^, ^18^, ^26^ trichomonas,^14^, ^29^ and herpes.^12^, ^18^, ^20^, ^24^ They differed from several studies^13^, ^42^ for gonorrhea and for syphilis.^43^, ^44^ Similar to our results, a large prospective study on health professionals was conducted to investigate the role of STIs and prostate cancer which did not find any association for neither gonorrhea nor syphilis.^17^ Extensive research has been dedicated to investigating the impact of prostatitis on the development of prostate cancer. A meta‐analysis of 16 studies revealed an odds ratio (OR) of 1.83 (95% confidence interval: 1.43–2.35), indicating a higher risk of developing prostate cancer among individuals with a history of prostatitis [54]. However, it is important to acknowledge that the possibility of a detection bias cannot be dismissed as a potential explanation for these results.^45^ Even though herpes zoster was studied the least among infections, two studies were consistent with our results.^30^, ^46^ On the contrary, two other studies with an HR = 1.42 (95% CI: 1.06–1.92) and HR = 1.97 (95% CI: 1.27–3.06) showed that having herpes zoster infection is a suggestive risk factor for later developing prostate cancer.^35^, ^36^ Hepatitis B and C have been studied in recent years and were inversely associated with prostate cancer,^21^, ^47^ while a cohort study showed that viral hepatitis of HCV and HBV combined were not associated with prostate cancer which was consistent with our study.^48^ The observed inconsistency in results may be attributed, in part, to the varying impact of racial differences among Latino, Black, and Caucasian populations where the rate of STD reporting is higher among Latinos and Africans in comparison to Caucasians.^18^ In addition, among individuals from diverse racial, ethnic, socioeconomic, or educational backgrounds, a history of gonorrhea may be indicative of multiple episodes or coinfections, which may result in a stronger association with prostate cancer risk.^43^ Another possible explanation for varying results is for example, in the case of gonorrhea, which often denotes a longer duration of untreated infection due to challenges in medical access, lack of proper or effective treatments, or even non‐compliance with treatment leading to prolonged inflammation. The frequency and timing of infection events can influence their overall effect on prostate cancer risk.

Within the literature, a significant body of evidence highlights intriguing associations between human papillomavirus (HPV), human immunodeficiency virus (HIV), and Mycoplasma infections, suggesting compelling connections that warrant further exploration. However, our EPICAP study did include data pertaining to these infections. While these infectious agents have garnered significant attention in the context of prostate cancer research, their inclusion in studies has yielded inconsistent results. Two meta‐analysis showed that the risk of prostate cancer increased when having a history of HPV or when being infected with certain species of mycoplasma.^39^, ^49^ On the contrary, for HIV infection, the results showed a decreased risk of prostate cancer, which could be explained by the altering testosterone levels in men with HIV.^50^

Moreover, and to our knowledge, there were no studies that specifically studied the role of Dengue or Polio or tuberculosis in the occurrence of prostate cancer.

Case identification was made in all private and public cancer centers in the department of Hérault. In 2011, the Hérault cancer registry observed 770 new cases of prostate cancer in men, of which 75% were less than 75 years old. Considering that the number of cases observed in 2012–2013 will be similar, approximately 1150 new cases were expected during the study period.^51^ The recruitment of cases was quite exhaustive since the identified eligible cases were 1098 over the study period, thus limiting the potential of having selection bias. Controls were randomly selected from the general population of the department of Hérault using quotas defined by age (5 years) to reflect the age distribution of the cases. Moreover, the SES distribution of the control group reflects the SES distribution of the entire department of Hérault to yield a control group similar to the general population of men to avoid selection bias. After the selection process, the distribution by SES between the control group and the general male population of the department of Hérault was compared. No significant difference was found, indicating that no major selection bias by SES had occurred in the control population.

Acknowledging the potential for recall bias in our study is essential, particularly when the data collection method relies on self‐reporting of exposure. This introduces the possibility of differential classification bias. In our case, the study involved self‐reporting a history of sexually transmitted infections (STIs). It is important to note that STIs are often stigmatized and can be asymptomatic, which may result in underestimation of their prevalence due to reluctance to answer, lack of awareness, or inadequate knowledge about the nature of the infection. However, the data collection was done face‐to‐face by a trained research clinical nurse using a standardized questionnaire and was carried out identically in both cases and controls. In addition, several questions regarding the variables of interest were also formulated differently multiple times, allowing cross‐referencing and thus limiting potential classification bias. It is noteworthy to consider the prevalence of controls, and in our study, the prevalence of controls was found to be 7.7% for gonorrhea. Comparatively, in the general population of France, the prevalence of gonorrhea among men aged greater than 45 is approximately 6% indicating a relatively similar prevalence, which helps minimize the potential impact of classification bias in our findings. However, we should consider that the prevalence of syphilis observed in EPICAP is around 1% which is lower than the prevalence of syphilis in the population of France which is around 4%^52^ indicating a potential underestimation of our results. When considering cases, we cannot entirely rule out the possibility of recall bias, potentially resulting in underreporting and subsequent underestimation of the true impact.

Our results remained unchanged after adjusting to non‐modifiable risk factors and major potential confounding variables such as level of education, physical activity, waist circumference, and non‐steroidal anti‐inflammatory drugs.

## 5 CONCLUSION

We found no association between sexually transmitted and non‐sexually transmitted infections in the occurrence of prostate cancer. Therefore, our results do not indicate an essential role for infectious agents in the etiology of prostate cancer. Due to the inconsistency in the results of previous studies, additional research is warranted to ascertain the role of inflammatory or infectious agents with a particular interest on sexually transmitted infections and the onset of prostate cancer to adjust and implement the proper preventive strategies to limit its occurrence.

## AUTHOR CONTRIBUTIONS

**Melissa Sawaya:** Formal analysis (lead); writing – original draft (lead); writing – review and editing (equal). **Emilie Cordina‐Duverger:** Data curation (equal); formal analysis (supporting); project administration (equal); resources (supporting); validation (supporting); writing – review and editing (supporting). **Pierre‐Jean Lamy:** Conceptualization (supporting); data curation (equal); funding acquisition (supporting); investigation (equal); project administration (supporting); writing – review and editing (supporting). **Xavier Rébillard:** Conceptualization (supporting); data curation (equal); funding acquisition (supporting); methodology (supporting); project administration (equal); writing – review and editing (supporting). **Brigitte Trétarre:** Conceptualization (supporting); data curation (equal); funding acquisition (supporting); investigation (equal); methodology (supporting); project administration (equal); writing – review and editing (equal). **Florence Menegaux:** Conceptualization (lead); data curation (lead); formal analysis (equal); funding acquisition (lead); investigation (lead); methodology (lead); project administration (lead); supervision (lead); validation (lead); writing – original draft (equal); writing – review and editing (lead).

## FUNDING INFORMATION

The EPICAP study was funded by Ligue nationale contre le cancer, Ligue contre le cancer du Val de Marne, Fondation de France, Agence nationale de sécurité sanitaire de l'alimentation, de l'environnement et du travail (ANSES).

The first author, Melissa SAWAYA, is funded by a 3‐year doctoral allowance from the Doctoral School of Public Health (EDSP), Paris‐Saclay University, for her PhD.

## CONFLICT OF INTEREST STATEMENT

All authors have no financial disclosures to report.

## ETHICAL APPROVAL

The study was approved by the Institutional Review Board of the French National Institute of Health and Medical Research (IRB‐Inserm n° 01–040 – November 2010) and by the French Data Protection Authority (CNIL N° 910,485 – April 2011).

## CONSENT TO PARTICIPATE

Each subject included in EPICAP signed a written informed consent.

## Data Availability

The datasets used and analyzed during the current study are available from the corresponding author upon reasonable request.
